# The Ontario printed educational message (OPEM) trial to narrow the evidence-practice gap with respect to prescribing practices of general and family physicians: a cluster randomized controlled trial, targeting the care of individuals with diabetes and hypertension in Ontario, Canada

**DOI:** 10.1186/1748-5908-2-37

**Published:** 2007-11-26

**Authors:** Merrick Zwarenstein, Janet E Hux, Diane Kelsall, Michael Paterson, Jeremy Grimshaw, Dave Davis, Andreas Laupacis, Michael Evans, Peter C Austin, Pamela M Slaughter, Susan K Shiller, Ruth Croxford, Karen Tu

**Affiliations:** 1 Institute for Clinical Evaluative Sciences, Toronto, Canada; 2 Clinical Epidemiology, Sunnybrook Health Sciences Centre, Toronto, Canada; 3 Faculty of Medicine, University of Toronto, Toronto, Canada; 4 Clinical Epidemiology Program, Ottawa Health Research Institute, Ottawa, Canada; 5 Faculty of Medicine, University of Ottawa, Ottawa, Canada; 6 Keenan research centre, Li Ka Shing Knowledge Instititue, St Michaels hopsital, Toronto, Canada; 7 Department of Public Health Sciences, University of Toronto, Toronto, Canada; 8 Department of Health Policy, Management and Evaluation, University of Toronto, Toronto, Canada; 9 Canada Research Chair in Health Knowledge Transfer and Uptake, Ottawa, Canada

## Abstract

**Background:**

There are gaps between what family practitioners do in clinical practice and the evidence-based ideal. The most commonly used strategy to narrow these gaps is the printed educational message (PEM); however, the attributes of successful printed educational messages and their overall effectiveness in changing physician practice are not clear. The current endeavor aims to determine whether such messages change prescribing quality in primary care practice, and whether these effects differ with the format of the message.

**Methods/design:**

The design is a large, simple, factorial, unblinded cluster-randomized controlled trial. PEMs will be distributed with ***informed***, a quarterly evidence-based synopsis of current clinical information produced by the Institute for Clinical Evaluative Sciences, Toronto, Canada, and will be sent to all eligible general and family practitioners in Ontario. There will be three replicates of the trial, with three different educational messages, each aimed at narrowing a specific evidence-practice gap as follows: 1) angiotensin-converting enzyme inhibitors, hypertension treatment, and cholesterol lowering agents for diabetes; 2) retinal screening for diabetes; and 3) diuretics for hypertension.

For each of the three replicates there will be three intervention groups. The first group will receive ***informed ***with an attached postcard-sized, short, directive "outsert." The second intervention group will receive ***informed ***with a two-page explanatory "insert" on the same topic. The third intervention group will receive ***informed***, with both the above-mentioned outsert and insert. The control group will receive ***informed ***only, without either an outsert or insert.

Routinely collected physician billing, prescription, and hospital data found in Ontario's administrative databases will be used to monitor pre-defined prescribing changes relevant and specific to each replicate, following delivery of the educational messages. Multi-level modeling will be used to study patterns in physician-prescribing quality over four quarters, before and after each of the three interventions. Subgroup analyses will be performed to assess the association between the characteristics of the physician's place of practice and target behaviours.

A further analysis of the immediate and delayed impacts of the PEMs will be performed using time-series analysis and interventional, auto-regressive, integrated moving average modeling.

**Trial registration number:**

Current controlled trial ISRCTN72772651.

## Background

There are gaps between the evidence-based, ideal clinical practice and what family practitioners (FPs) actually do. We understand very little about the reasons for these gaps [1]. Printed educational messages (PEMs), ranging from short directive statements to long guidelines, have often been used in the hope of narrowing these gaps [2], but there is profound uncertainty around their usefulness [3-5]. PEMs fell into disfavour among researchers in this field after a 1997 systematic review found their effects to be uncertain [6]. A recent larger overview found a median absolute improvement of 8% (range 4–17%) [7].

However, no review can overcome the limitations of the primary evidence: the small number of trials (each one of which is small in size), and their continued methodological weaknesses (insufficient power to detect modest effects, and unit of analysis errors).

## Aims and objectives

1. To conduct a large, reliable and relevant (pragmatic), randomized controlled trial of the effect of printed educational messages (PEMs) on guideline adherence among Ontario primary care physicians.

2. To determine whether there is any difference in impact on physician guideline adherence between: 1) a postcard-sized, short, directive message, 2) a two-page, longer, explanatory message, or 3) a combination of both.

3. To determine the relative effectiveness of PEMs in narrowing gaps for different health problems.

A randomised trial will help define the role of PEMs, while increasing our understanding of the process of implementing evidence of effectiveness into daily clinical care [8,9].(In Canadian research, the word 'translation' is used, in place of 'implementation'.)

## Methods/design

Pragmatic, randomized controlled trials are reliable evaluations of the effectiveness of often complex health care interventions carried out under real-world conditions [8,9].

Effectiveness of the intervention usually is assessed by impact on simple outcomes of importance to users of the intervention, such as: death, disability, user satisfaction, utilization of care, and quality of life. Pragmatic trials are attuned to the same criteria of effectiveness as those used by policymakers – important and visible outcomes in usual health service planning entities, and under typical service limitations. Pragmatic trials take into account the varied ways that interventions are implemented in the real world. In contrast, classic explanatory randomized trials (efficacy trials) test the effects of an intervention, often a drug, under idealised and tightly controlled conditions, and on narrowly defined groups of individual patients. Classic efficacy trials usually are carried out to advance medical knowledge, whereas pragmatic trials are concerned with assessing the effects of interventions as they are usually used, in typical settings, and on typical users.

### Choice of target physician practices

We used several criteria to identify evidence/practice gaps important in primary care: the gap involves a common disorder in Canadian primary care; evidence-based practice is not constrained by structural, financial or other barriers; the evidence/practice gap is large and may cause patient harm or unnecessary cost; well-tested process indicators exist that are measurable using administrative datasets; and the gaps span a range of clinical practice behaviours.

As a result, we selected three evidence-practice gaps leading to clinical choices that family doctors should make more often. These gaps are based upon Ontario administrative data.

1. Prescription of angiotensin-converting enzyme (ACE) inhibitors, aggressive hypertension treatment, and cholesterol-lowering treatment for persons with diabetes (each falls short of guideline targets by at least 30%) [10-33].

2. Referral of persons with diabetes for eye screening by an optometrist or ophthalmologist (50% below guideline-recommended levels) [34-40].

3. Prescription of diuretics as first-line therapy for newly diagnosed hypertension (~40% below guideline-recommended levels) [41-50].

### Trail intervention: the printed educational messages

We will develop PEMs to address each of the three identified evidence/practice gaps, using input from a diverse group of physicians to identify barriers to evidence-based practice and from a communications consultant to inform design of the messages [51].

The PEMs will be distributed along with ***informed***, a peer-reviewed, evidence-based practice synopsis, which has been mailed quarterly since 1994 to nearly 15,000 primary care providers in Ontario. Subscription is voluntary and is at no cost to the subscriber. Articles for each eight-page issue are developed by clinical and research staff from the Institute for Clinical Evaluative Sciences (ICES), Toronto, Canada.

Two PEMs will be developed for each of the three evidence/practice gaps:

• A short, directive, evidence-based PEM on a postcard-sized card (referred to as an outsert) stapled to the lower-left quarter of the front page of ***informed***.

• A longer two-page insert (indistinguishable from the rest of the periodical in size, style and editing) focusing on the same topic as the outsert, but excluding the directive statements and including more background, evidence-based guidelines and references.

### Research design

**Figure 1:**
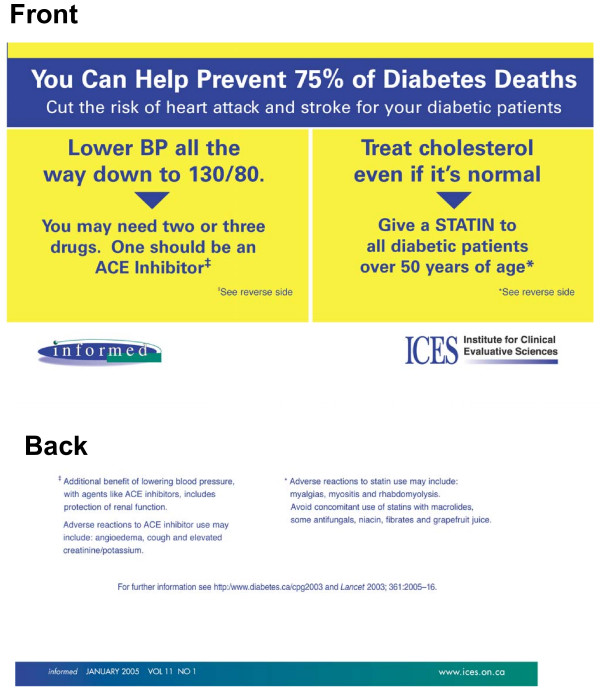
Printed educational message "outsert" for replicate 1.

**Figure 2:**
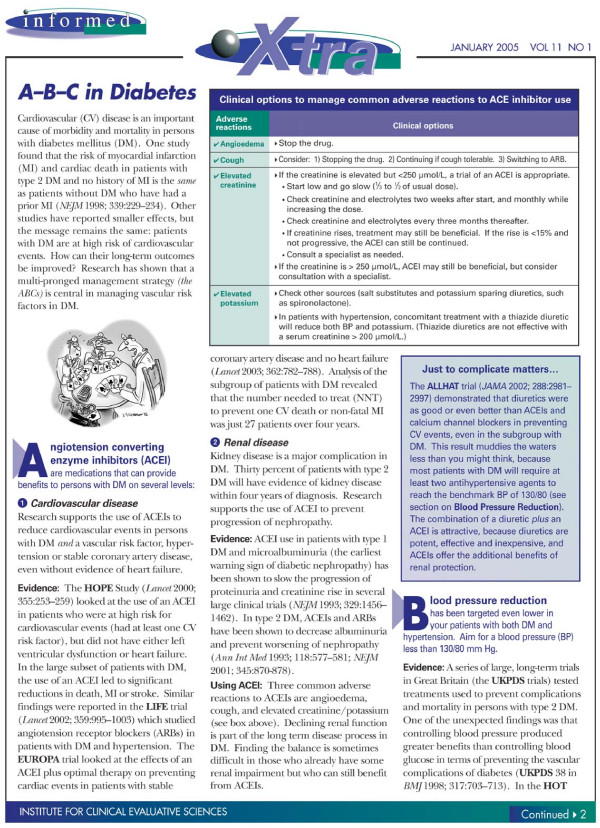
Printed Educational Message "Insert" for Replicate 1 (part 1).

**Figure 3:**
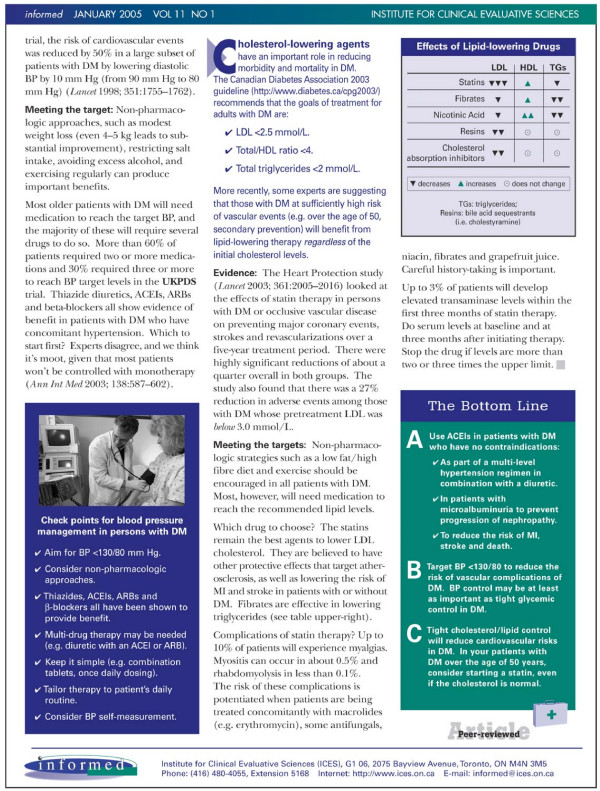
Printed Educational Message "Insert" for Replicate 1 (part 2).

The research design is a factorial, cluster-randomized controlled trial with three replicates [52,53]. ***informed ***is published four times per year. We will conduct three replicates of the trial to cover the three evidence-practice gaps over a nine-month period (three successive mailouts of ***informed***). We will test the effects of short (directive) and long (discursive) PEMs compared with no PEM on the clinical practices of primary care physicians, and on related patient outcomes. Table 1 describes the intervention groups for each of the three replicates. In the first replicate (ACE inhibitors, hypertension treatment, and cholesterol-lowering agents for diabetes), the first intervention group will receive a copy of ***informed ***with both the short, directive, evidence-based outsert stapled to the lower-left quarter of the front page, and the longer two-page insert focusing on the same topic as the outsert. The second intervention group will receive the identical ***informed***, with only the above-mentioned outsert. The third intervention group will receive the identical copy of ***informed ***with the above-mentioned insert. The control group will receive the identical ***informed ***only, without the insert or the outsert. The health care topic shared by the insert and outsert will not be covered elsewhere in that issue of ***informed***. The outsert for this first replicate is shown in Figure 1 and the insert in Figures 2 and 3.

**Table 1:** Description of the intervention groups within each of the three replicates

***REPLICATE 1: Assertive hypertension and cholesterol treatment in patients with Diabetes***			
		**INSERT**	**No INSERT**
**OUTSERT**		1. Insert & Outsert	2. Outsert only
**NO OUTSERT**		3. Insert Only	4. No PEM
			
***REPLICATE 2: Retinal Screening for Patients with Diabetes***			
		**Insert**	**No insert**

**OUTSERT**	*Patient Reminder Note*	1. Insert & Outsert & Patient Reminder	2. Outsert & Patient Reminder Note
	*No Patient Reminder Note*	3. Insert & Outsert	4. Outsert only
**NO OUTSERT**		5. Insert Only	6. No PEM
			
***REPLICATE 3: Diuretics for first-line treatment of hypertension***			
		**Insert**	**No insert**

**OUTSERT**	*Theory-based Outsert*	1. Insert & Theory-based Outsert	2. Theory-based Outsert Only
	*Non-theory-based outsert*	3. Insert & Non-theory-based Outsert	4. Non-theory-based Outsert only
**NO OUTSERT**		5. Insert Only	6. No PEM

For the second replicate (retinal screening in patients with diabetes), in addition to the short, directive outsert and the longer, explanatory insert, we will add a reminder note that physicians could give to their patients to supplement the verbal reminder that we encourage physicians to give. Because it is not clear whether this patient-held reminder to make an appointment with their eye care provider is any more effective than the verbal reminder that we will encourage physicians to give, we will randomize those physicians receiving an outsert to receive a pad of the patient-aimed reminder slips.

For the third replicate (using thiazides as first-line treatment for hypertension), we will use *two *different short-directive outsert messages (in addition to the long, explanatory insert message). Our OPEM team will develop the first outsert message, whereas a team of psychologists with experience in knowledge implementation and the use of psychological theories will develop the second outsert message. With the addition of a theory-based outsert, we will be able to determine whether a message that is based on psychological theory, specifically on the *Theory of Planned Behaviour*, will be more effective in changing clinical behaviour toward more evidence-based practice than a message that is based on 'standard' methods, which are uninformed by an explicit theoretical basis[54]. (See "Ancillary Studies" below.)

### Allocating participants to trial groups

Trials of interventions aimed at changing clinical practices must be randomized at the level at which they are directed – that is, at the level of the doctor, not his or her patients. In group practices, however, doctors may well share information. To prevent contamination, randomization must take place at the level of the group rather than the individual practitioner. A group practice is defined here as all primary care physicians who share a common street address, or who share a common office number in a multi-office building. The eligible Ontario primary care doctors will be fee-for-service family physicians (FPs) and general practitioners (GPs) (Ontario Health Insurance Program [OHIP] billing code 00).

These practitioners will be identified from the health care providers database held at ICES. They will be verified to be in active practice. In order to enable prescribing outcomes to be measured, we will ensure that they have an adequate volume of seniors among their patients by requiring that they write at least 100 prescriptions appearing in the ODB (Ontario Drug Benefit Program) database in the year prior to enrollment. The physician identifier obtained from these ODB claims first will be linked to the physician's College of Physicians and Surgeons of Ontario (CPSO) number at ICES. Addresses of practitioners will be obtained by hand-linking the CPSO number to the CPSO database, which is publicly available on the Internet. The CPSO website can be queried by CPSO number and will supply physician name and practice address.

All FPs and some GPs are already on the ***informed ***mailing list. Those who are not currently on the list will be added for the three intervention issues of ***informed***. This list will be sorted by addresses of practitioners into practices, in order to prevent practitioners with the same address, who may share administrative staff or communicate readily with each other, from being randomized to different groups (i.e., to reduce contamination). Using a computer-generated sequence of random numbers, the practices will then be randomized to one of the intervention groups. For each of the three replicates, we will re-randomize physician practices to one of the intervention groups.

The relevant patients for each practice are defined as any patient attending that practice who meets study entry criteria. For each of the gaps, we are using a different algorithm to identify relevant patients (see Table 2). A patient may be counted multiple times, as they are free to attend more than one primary care practice. This approach is conservative, and gives all physicians "credit" for appropriate prescribing or referral decisions made by any of the physicians who saw that patient during the study period. In a sensitivity analysis, we will use a more rigorous rule for defining physician-patient relationships, whereby patients will be assigned only to the primary care provider with whom they had a plurality of their visits. In a tie, the patient will be assigned to the primary care physician who wrote the greatest number of their prescriptions during the study period. Patients who still cannot be unambiguously assigned will be allocated randomly to one of the practitioners who have written prescriptions for them during the study period.

**Table 2:** Patient population and primary outcome for each of 3 replicates

**Replicate**	**Evidence/Practice Gap**	**Patients studied**	**Expected patient N**	**Primary Outcome**
1	Use of ACE inhibitors, blood pressure and cholesterol-reducing drugs (ABCs); each fall short of guideline targets by at least 30%	All prevalent cases of persons with diabetes, aged ≥ 65 years	280,000	% of target patients receiving an ACE inhibitor, % receiving 2 or more antihypertensive, and % receiving lipid-lowering drug
2	Retinopathy screening in incident Type 2 DM; 50% below guideline-recommended levels	All incident cases of persons with diabetes, aged >30 years	100,000	% of target patients receiving complete eye exam from optometrist or ophthalmologist
3	Use of hydrochlorothiazide (HCTZ) as initial therapy in uncomplicated hypertension; ~40% below guideline-recommended levels	All patients ≥ 65 years, newly initiated on antihypertensive therapy	30,000	% of target patients receiving a diuretic

The Registered Persons Database (RPDB) identifies all persons in the province who are eligible for OHIP coverage. It will be used to link patient participants to the administrative databases, and to detect death and out-migration of patients over the course of the study. Claims from the ODB Program will be used to measure the prescribing changes that result from the interventions. These data are available only for people 65 years of age and older. Accordingly, the two drug-related replicates are restricted to that age group of the Ontario population. The ODB data are delivered to ICES within two weeks of the end of the month of the claim. Claims data from OHIP will be used to identify physician patient linkages, and to measure the delivery of ophthalmologic and optometric screening. All of these datasets are available at ICES, and all bear encrypted health card numbers that are unique to patients but common over time and across data sets.

### Outcome measurement

Follow-up by means of routine data will cover four quarters before and after each prescribing-related PEM intervention. Each of the process-of-care measures has been used in large-scale studies using databases held at ICES.

### Proposed sample size

We wished to have adequate power to detect modest changes in the way physicians practice. Based on the median absolute effectiveness of PEMs estimated from the Grimshaw review (8%) [7], it would be important to detect 5% and 10% absolute improvements in our target behaviours. Even 5% improvements in the prevalence of evidence-based care for these common conditions will be an important achievement, with significant health benefits for many Ontarians.

In pilot data, the baseline-prescribing rate of ACE inhibitors in persons with diabetes was 36%, and the intra-class correlation for ACE inhibitor use by patients clustered within physicians was 9.2%. With an assumption that one-half of doctors work in solo practices, we ran these data testing power at a mean of three patients and ten patients per practice. Monte Carlo simulations demonstrated that a trial with 1250 practices per arm, with 10 patients per practice and an alpha of 0.05 will provide more than 98% power to detect a 10% absolute increase in the rate of ACE inhibitor use, more than 97% power to detect a 5% absolute increase, and more than 98% power to distinguish between the effects of the combined intervention and either alone, assuming the combined effect to be additive.

Because the baseline rates of the other interventions are comparable or lower (screening for retinopathy, diuretic use, aggressive blood pressure-reducing, and cholesterol-lowering therapies), and the number of patients per practitioner with these conditions is the same or higher, we will have adequate power to detect similar absolute increases for those endpoints.

Later, with the addition (see Table 1) of two groups in the second (patient reminder note) and third (theory-based outsert) replicates, a less comprehensive, but approximate simulation suggested that the impact on power would be minor and would most certainly be greater than the often used target of 80%. The research opportunity that this modification presented was judged to outweigh the likely loss of power. Therefore, we maintained 1,250 practices in the two groups that did not receive an outsert with ***informed***, and assigned 625 practices to each of the four groups who were sent an outsert.

### Recruitment, compliance, loss to follow-up, and drop-out

Practitioners may of course decide to ignore the PEMs or never open the envelope containing ***informed***. We will not know what practitioners will do with the intervention, a normal feature of the pragmatic trial philosophy that tests interventions under real-world conditions. ***informed ***will be mailed in the usual fashion to all identified practitioners. Any practitioners who subsequently remove themselves from the mailing list for ***informed ***will be analysed in the allocated arm on an intention-to-treat basis.

While the unit of analysis is the physician, loss of patients is important to detect in order to maintain accurate denominators for the determination of outcome rates. Patients may be lost due to removal from the system (death or out-migration), or change of provider. The former will be detected in the RPDB, the latter by a plurality rule for prescriptions. For the baseline analysis (pre-intervention period), the initially assigned patients for each physician will be used. For each of the succeeding observation periods (three pre- and four post-intervention), initial patients will be confirmed by eliminating patients no longer eligible according to the RPDB, and ensuring that assigned patients are still being cared for by the study physician through a requirement that the study physician must have written the plurality of the patient's prescriptions in that period.

### Analyses

We hypothesize that the active intervention arms will be superior to control, similarly effective to each other, and with some additive effect when both are delivered. Analyses will be concentrated on this hypothesis.

The study employs a cluster randomized design. The analysis will be carried out using multi-level, hierarchical, logistic regression models, with the trial arm being a physician-level variable, and the prescribing (or screening) outcome a patient-level variable. These models account for clustering of patients within practices, and the consequent lack of independence of prescribing outcomes within a given physician's place of practice [55]. No place-of-practice characteristics, aside from the arm of the trial and baseline adherence to guidelines, will be entered into the model in the primary analysis. With so many physicians randomized to each arm, we anticipate that measured and unmeasured physician factors that might affect the outcome will be equally distributed between the arms. Moreover, including more place-of-practice characteristics in the model would reduce the statistical power to detect the primary outcome.

### Subgroup analyses

Failure to consistently implement evidence into improved prescribing decisions has been associated with older physicians and those without specialty certification [56]. While we would like to test the impact of certification, no data exists describing this status that can be linked to our datasets.

Evidence-practice gaps also have been associated with physician reimbursement arrangements, patient volumes, physician gender, site and extent of training, group size, and work load [57]. Thus, we plan subgroup analyses of male versus female physicians, location of practice (urban or rural), practice volume, solo versus group practice, and years since graduation. As before, multilevel, logistic regression models will be used to assess the association between place-of-practice characteristics and target behaviours.

A further analysis of the immediate and delayed impacts of the printed educational material will be performed using time-series analysis and interventional, autoregressive, integrated moving average (ARIMA) modeling [58]. The group to which the patient's practitioner was randomized will be included as a covariate. An immediate change will be defined as a significant shift in prescribing patterns from projected estimates within four months of the intervention. Delayed effects will be measured with simple time-series analysis, such as exponential smoothing and ARIMA models by comparing actual with predicted utilization estimates. Stationarity will be assessed using the autocorrelation function and the augmented Dickey-Fuller test [59]. The auto-correlation, partial autocorrelation, and inverse autocorrelation functions will be assessed for model parameter appropriateness and seasonality. The presence of white noise will be assessed by examining the autocorrelations at various lags using the Ljung-Box test [60].

The question of whether there are differences in response to PEMs for different diseases patient health status levels and health care problems is addressed in the replicates. Differences between these replicates will be discussed, and will not *a priori *be subjected to meta-analysis. In the event of substantial homogeneity of outcomes, we will consider meta-analysis across the replicates.

There will be one analysis for each replicate upon receipt of one year of follow-up data.

### Ancillary studies

Two smaller studies will be conducted in conjunction with the OPEM study. Both will take advantage of the infrastructure, which will already be in place for the main study to obtain additional, related information about PEMs – namely information about the causal mechanisms through which PEMs lead to behaviour changes.

The Testing a TheoRY-informed Message (TRY-ME) study will be associated with the third intervention (prescription of diuretics as first-line therapy for newly diagnosed hypertension) [64]. Two outserts will be developed. The wording of the first outsert containing a 'standard' message will be developed by a team of clinical researchers experienced in implementation research and in the development of short educational messages directed to clinicians. The wording of the second, theory-based message will be developed by a team of psychologists with experience in implementation research, and clinical researchers experienced in the use of psychological theories. Otherwise, the two messages will use similar styles, font sizes and colours. As indicated in the above "Proposed Sample Size" sub-section, the OPEM study will retain sufficient power for the primary outcomes, while also permitting a comparison of 'standard' and theory-based outserts.

The interpretation of the results of the OPEM trial and assessment of its likely generalisability would be enhanced if we had additional information about the causal mechanisms through which the interventions worked, and how these were modified in the presence of different barriers and enablers. The second ancillary study will conduct a theory-based process evaluation alongside the OPEM trail. "Looking inside the black box: a theory-based process evaluation alongside a randomised controlled trial of printed educational materials" [65] will develop surveys, based upon the *Theory of Planned Behaviour *[56], for the second and third OPEM interventions, and survey a sub-sample of recipients from each arm of the trial two months before and six months after the dissemination of the relevant edition of ***informed***.

### Ethical approval

This study has received approval from the Research Ethics Board at Sunnybrook and Women's College Health Sciences Centre. The data linkage processes described above have been used at ICES previously, and meet the requirements of the ICES research agreement with the Ontario Ministry of Health and Long-Term Care, under which ICES has access to the administrative data.

## Discussion

We understand very little about the reasons for the gaps between what family practitioners do in clinical practice, and what the evidence-based ideal says they should do. The simplest intervention to narrow these gaps is the PEM, ranging from a one-sentence directive to a long guideline; and they have often been used, but in an intuitive fashion, with neither an underlying theory of behaviour change nor a deep understanding of physicians' barriers to change.

After a 1997 systematic review found their effects to be uncertain, PEMs fell into disfavour. However, a more recent and larger overview found a median improvement of 8% in comparison with no PEM – a result which was similar in effect to more expensive and intensive strategies. Nevertheless, this review cannot overcome the limitations of the primary evidence: the small number of trials (each one of which is small in size), and their continued methodological weaknesses (insufficient power to detect modest effects, and unit of analysis errors). More evidence is needed in order to decide whether PEMs can be used to help change clinical practice.

We have proposed a trial that is designed to address these shortcomings. The large size of the trial provides enough power to confidently and precisely delineate modest effects. Modest effects are important, given the large absolute impact that is achievable when the intervention is applied to a common health problem. By randomizing at the level of the physician place-of-practice, the trial avoids contamination and unit of analysis error. In addition to answering the question of whether PEMs change primary care practitioners' therapeutic decisions, this study, along with its two ancillary studies, also will compare the impact of two types of PEMs (the short, directive versus a two-page descriptive message), and will examine the contextual and sociological factors (e.g., health problems, location, style of physician practice and duration of practice) which may influence the impact of PEMs.

If passive dissemination of PEMs is shown to be effective, this study may give professional, administrative, and patient organizations interested in change toward evidence-based practice the impetus to develop systematic programs for practice change using PEMs. If shown to be ineffective, use of PEMs as a single practice change intervention would be discouraged, thereby eliminating waste and turning research attention toward other interventions, including a possible role for PEMs as one element in a multi-faceted intervention. If effectiveness is shown to vary by disease problem, barrier, subgroup of practitioner, or any other factor, this study will help inform how different kinds of PEMs should be used in different situations and health problems.

While no economic analysis has been proposed, pending a determination of the effectiveness of PEMs in changing practice, we will collect all costs attributable solely to PEM development, design and dissemination. If the intervention is effective, we will work to set up an initial cost analysis, in which the health care utilization and prescribing changes, if any, of the intervention will be assessed in view of the marginal costs of the PEM intervention. We also will approach professional bodies and patient advocacy groups to offer help in developing PEM strategies for health care quality improvement.

## Abbreviations

ACE – Angiotensin Converting Enzyme

ARIMA – Auto-Regressive Integrated Moving Average

CFPC – College of Family Physicians of Canada

CIHI – Canadian Institute for Health Information

CPSO – College of Physicians and Surgeons of Ontario

FP – Family Physician

GP – General Practitioner

ICES – Institute for Clinical Evaluative Sciences

ODB – Ontario Drug Benefit Program

OHIP – Ontario Health Insurance Plan

OPEM – Ontario Printed Educational Message

PEM – Printed Educational Message

RPDB – Registered Persons Database

TRY-ME – TheoRY-based MEssage

## Competing interests

The author(s) declare that they have no competing interests.

## Authors' contributions

All authors contributed to the development of the study.

All authors read and approved the final manuscript.
